# Comparative genomics of non-pseudomonal bacterial species colonising paediatric cystic fibrosis patients

**DOI:** 10.7717/peerj.1223

**Published:** 2015-09-15

**Authors:** Kate L. Ormerod, Narelle M. George, James A. Fraser, Claire Wainwright, Philip Hugenholtz

**Affiliations:** 1 Australian Infectious Diseases Research Centre, School of Chemistry and Molecular Biosciences, University of Queensland, Brisbane, Queensland, Australia; 2 Health Support Queensland, Department of Health, Queensland Government, Herston, Queensland, Australia; 3 School of Medicine, University of Queensland, Brisbane, Queensland, Australia; 4 Department of Respiratory and Sleep Medicine, Lady Cilento Children’s Hospital, South Brisbane, Queensland, Australia; 5 Queensland Children’s Medical Research Insitute, Herston, Queensland, Australia; 6 Australian Centre for Ecogenomics, School of Chemistry and Molecular Biosciences, University of Queensland, Brisbane, Queensland, Australia

**Keywords:** Comparative genomics, Cystic fibrosis, Microevolution, Antibiotic resistance, Lateral gene transfer, Host adaptation, Serial isolates

## Abstract

The genetic disorder cystic fibrosis is a life-limiting condition affecting ∼70,000 people worldwide. Targeted, early, treatment of the dominant infecting species, *Pseudomonas aeruginosa*, has improved patient outcomes; however, there is concern that other species are now stepping in to take its place. In addition, the necessarily long-term antibiotic therapy received by these patients may be providing a suitable environment for the emergence of antibiotic resistance. To investigate these issues, we employed whole-genome sequencing of 28 non-Pseudomonas bacterial strains isolated from three paediatric patients. We did not find any trend of increasing antibiotic resistance (either by mutation or lateral gene transfer) in these isolates in comparison with other examples of the same species. In addition, each isolate contained a virulence gene repertoire that was similar to other examples of the relevant species. These results support the impaired clearance of the CF lung not demanding extensive virulence for survival in this habitat. By analysing serial isolates of the same species we uncovered several examples of strain persistence. The same strain of *Staphylococcus aureus* persisted for nearly a year, despite administration of antibiotics to which it was shown to be sensitive. This is consistent with previous studies showing antibiotic therapy to be inadequate in cystic fibrosis patients, which may also explain the lack of increasing antibiotic resistance over time. Serial isolates of two naturally multi-drug resistant organisms, *Achromobacter xylosoxidans* and *Stenotrophomonas maltophilia*, revealed that while all *S. maltophilia* strains were unique, *A. xylosoxidans* persisted for nearly five years, making this a species of particular concern. The data generated by this study will assist in developing an understanding of the non-Pseudomonas species associated with cystic fibrosis.

## Introduction

The impaired clearance of the respiratory tract of patients with cystic fibrosis (CF) permits chronic infection by microorganisms (reviewed in Boucher, 2007). *Pseudomonas aeruginosa* and *Staphylococcus aureus* are the two most commonly isolated species (Emerson et al., 2010), with prevalence rates over 50% (Razvi et al., 2009). *Haemophilus influenzae* is isolated in ∼20% of cases (Razvi et al., 2009; Cardines et al., 2012). The species present in CF patients has been observed to fluctuate with age; *S. aureus* and *H. influenzae* are more often associated with younger patients, while *Stenotrophomonas maltophilia* and *P. aeruginosa* increase in prevalence in older patients (Razvi et al., 2009; Cox et al., 2010). *S. aureus* is the most commonly isolated organism in children under 10 years, colonising over 60% of patients (Razvi et al., 2009). *P. aeruginosa* becomes the dominant species isolated from adult patients, isolated from ∼80% of patients (Razvi et al., 2009). *P. aeruginosa* has previously been identified as the primary disease causing agent (Høiby, 1982) and improved patient outcomes have been achieved through aggressive antibiotic therapy targeting this species during the early stages of disease prior to the establishment of chronic infection (Høiby, Frederiksen & Pressler, 2005). Life expectancy of CF patients has improved in recent years, however still remains low at ∼40 years (Quon & Aitken, 2012).

Of current concern is the possibility that antibiotic treatment specific to *P. aeruginosa* may be increasing the prevalence and diversity of non-*Pseudomonas* bacteria, associated with the CF lung with unknown consequences (Hansen, 2012). The prevalence of *S. aureus*, *S. maltophilia* and *Achromobacter xylosoxidans* have all been reported to have increased in recent years (Emerson et al., 2010). A study of German CF centres found a 20% prevalence of multiple emerging species, including *S. maltophilia*, *Achromobacter* spp., *Klebsiella* spp., *Burkholderia cepacia* complex or multiresistant *S. aureus*, in adults compared with 10% in children and adolescents (Steinkamp et al., 2005). Of the patients testing positive for these bacteria, 92% had been administered antibiotics during the six month study period compared with 53% of total participants (Steinkamp et al., 2005). In addition, the development of culture-independent detection methods has established the polymicrobial nature of CF including the identification of species previously not associated with CF including *Prevotella denticola*, and members of the families *Rickettsiaceae* and *Coxiellaceae* (Rogers et al., 2004; Harris et al., 2007; Cox et al., 2010; Van der Gast et al., 2011). These methods have also exposed a greater degree of microbial community heterogeneity between CF patients and also a greater degree of fluctuation over time than was previously appreciated from culture-based methods (Lim et al., 2014).

The recognition of CF as a polymicrobial condition has complicated traditional views of the progression of this disease. In polymicrobial conditions, the presence of some species may only be made possible by the presence of others and the clinical consequences associated with the presence of some species may also be altered by the presence of others (reviewed in Brogden, Guthmiller & Taylor, 2005). In CF, *P. aeruginosa* is associated with an increased likelihood of anaerobes such as *Prevotella* and *Veillonella* (Tunney et al., 2008). However, their identification was not associated with decreased lung function or increased frequency of exacerbation (Tunney et al., 2008). In fact, the role of many of the less common bacteria in CF in terms of clinical significance remains unknown (reviewed in Sibley, Rabin & Surette, 2006). Further to this, a decrease in lung function has been associated with decreasing microbial diversity (Van der Gast et al., 2011) indicating diversity may be a sign of a healthier lung environment. Some of the anaerobes detected in CF patients are also detected in healthy volunteers, although in significantly lower numbers (Tunney et al., 2008). While the lung was previously considered a sterile environment, the advance of culture independent techniques has shown this is not the case (Dickson, Erb-Downward & Huffnagle, 2013), further complicating the investigation of the pathogenesis of CF. Thus, there is a need to further investigate the characteristics of the specific strains present in the CF lung to determine if they are in some way unique in comparison to other examples of the same species, as this will help determine their relevance in the clinical context.

As a chronic condition requiring long-term antibiotic use, CF is further complicated by microvariation seen in persisting species that both adapts them to the host environment and can lead to the emergence of antibiotic resistance. This variation has been characterised in *P. aeruginosa* where, over an eight year infection, virulence factors were selected against, likely due to their immunogenic properties (Smith et al., 2006). In *S. aureus* also, key strategic characteristics such as the production of a polysaccharide capsule and the formation of biofilms commonly differ between early and late stage isolates from continuously colonised patients (Hirschhausen et al., 2013). Antibiotic resistance has been described in multiple *P. aeruginosa* isolates of the same lineage infecting different patients, attributed to unique genetic changes in each strain (Yang et al., 2011). This indicates that multiple avenues are available to an infecting species to respond to similar environmental pressures, however, our knowledge of which avenues are used by non-*Pseudomonas* species is limited.

We undertook comparative genome analysis of non-*Pseudomonas* isolates from three paediatric CF patients looking for characteristics that may have facilitated their colonisation or indicate their clinical significance. We also sought evidence that they may be being selected for by antibiotic treatment. We included both typical species and those that are increasing in prevalence in CF patients with a particular focus on cases where persistent colonisation was observed across multiple time points. By linking genome data with patient treatment data we have been able to investigate the impact of antibiotic treatment and found evidence that susceptible organisms, either due to lack of penetration of the antibiotic or protection by other species, were surviving throughout treatment periods. Both the antibiotic resistance and virulence gene arsenal of the investigated isolates did not differ substantially from previously analysed examples of the same species, which is in agreement with the nature of the CF lung promoting chronic bacterial colonisation. We also observed strain evolution occurring during persistent colonisation, both via SNPs and through the movement of mobile elements, the latter being an isolated but significant case of acquisition of antibiotic resistance genes.

## Methods

### Sample collection and quantification

Bronchoalveolar lavage (BAL) samples were collected as part of the Australasian Cystic Fibrosis BAL Study that was approved by the Royal Children’s Hospital Ethics Committee RCH HREC PROTOCOL NO.: 1998/001. Informed consent was obtained from the children’s parents for sample collection, storage and subsequent testing. All BAL samples were collected under general anaesthesia using standard procedures. Five ten-fold serial dilutions from 500 µL of BAL fluid (BALF) were used for quantitative colony counts. 100 µL of undiluted BALF and each dilution were plated onto six different selective media: horse blood (bioMérieux, Marcy-l’Étoile, France), mannitol salt (BD Biosciences, San Jose, California, USA), MacConkey (bioMérieux, Marcy-l’Étoile, France), chocolate bacitracin (CBA, Oxoid, UK), cetrimide (Oxoid, UK) and *Burkholderia cepacia* (Mast Group Ltd, Bootle, Merseyside, UK) agar. For sputum samples, 10 µL of the undiluted sample was plated directly onto selective media plates. Plates were then incubated at 37 °C (5% CO_2_ for CBA) and read at 24 and 48 h. The colony count was determined from plates bearing between 30 and 100 individual colonies. The final count is expressed as colony forming units per ml of BALF for each bacterial colony type of interest. Colony type identification tests were carried out as required: API 20 NE (bioMérieux, France, non-fastidious, non-enteric Gram-negative rods), VITEK GN-ID (bioMérieux, France, Gram-negative bacilli), coagulase production and latex agglutination (*S. aureus*), optochin susceptibility (*Streptococcus pneumoniae*) and X/V growth factor requirement (*Haemophilus*).

### Purification and antibiotic susceptibility testing

Between 1 and 4 colonies from selective media plates of each sampled species were diluted to 0.5 McFarland standard and then replated, cultured and stored at −80 °C. This same dilution was used as the inoculum for antibiotic susceptibility testing: Clinical and Laboratory Standards Institute (CLSI) disk diffusion testing for *Pseudomonas*, *Streptococcus* and *Haemophilus* and VITEK (bioMérieux, France) instrument analysis and comparison with CLSI minimum inhibitory concentration breakpoints for other species.

### DNA extraction and sequencing

DNA was extracted from pure cultures of each selected isolate using the PowerSoil DNA isolation kit (MO BIO Laboratories Inc, Carlsbad, California, USA). Indexed whole-genome sequencing libraries were prepared with the Nextera DNA sample preparation kit (Illumina, San Diego, California, USA). Libraries were pooled such that each isolate was approximately equal in concentration. This pool of libraries was submitted to the Queensland Centre for Medical Genomics (University of Queensland, Australia), and half a lane of 2 × 100-bp paired-end data was generated on an Illumina HiSeq2000 sequencer.

### Draft genome assembly

Reads were trimmed using Trimmomatic v0.30 (Bolger, Lohse & Usadel, 2014) and assembled using SOAP de novo v1.05 (Luo et al., 2012). Contigs were ordered against reference genomes using CONTIGuator v2.7.3 (Galardini et al., 2011) and gaps were closed where possible using IMAGE (Tsai, Otto & Berriman, 2010) within the PAGIT wrapper v1.64 (Swain et al., 2012). Genomes were submitted to RAST for annotation (Aziz et al., 2008). Draft assemblies were compared with reference genomes using progressiveMauve (Darling, Mau & Perna, 2010). Contamination and completeness of each draft assembly were estimated using CheckM v0.9.7 (Parks et al., 2015). ANI was calculated using ANI calculator (http://enve-omics.ce.gatech.edu/ani/index, last accessed 2 June 2015).

For isolates of the same species, each strain was also mapped to a reference genome selected based on the lowest percentage of unmapped reads. Mapping to reference genomes was performed using BWA 0.6.2-r126 (Li & Durbin, 2009) using default settings. Duplicate reads were marked using Picard 1.41 (http://www.picard.sourceforge.net) and base quality score recalibration and realignment around indels was performed using GATK 2.4-9-g532efad (McKenna et al., 2010). SNPs and indels were identified using GATK 2.4-9-g532efad following best practice guidelines (DePristo et al., 2011). Larger variation was detected using Breakdancer 1.1.2 (Chen et al., 2009) and CREST (Wang et al., 2011). Mappings were visualised and SNP associated amino acid changes were determined using CLC Genomics Workbench (Qiagen, Velno, Netherlands). Unmapped reads were collected using SAMtools (Li et al., 2009) and regions of zero coverage were identified using BEDTools (Quinlan & Hall, 2010).

### Comparative genome analysis

Antibiotic resistance and virulence related genes were identified using BLAST with the Comprehensive Antibiotic Research Database (McArthur et al., 2013) and the Virulence Factor Database (Chen et al., 2012) in addition to examining RAST output and manual comparison with various annotated reference genomes. For comparison with the CARD, a cut off of ≥40% identity over ≥60% of the query sequence was required. For comparison with the VFDB, a cut off of over 60% coverage of the target and over 80% identity was required. BLAST was used to identify candidate instances of horizontal gene transfer using a cut off of ≥95% over 500 bp. Genomic islands and prophage regions were detected using Island Viewer (Dhillon et al., 2013) and PHAST (Zhou et al., 2011). Maximum likelihood “genome” trees were inferred using MEGA (Tamura et al., 2013) with 500 bootstrap replicates based on concatenated alignments of 83 single copy marker genes from isolates and reference strains using CheckM (Parks et al., 2015).

## Results and Discussion

**Figure 1:**
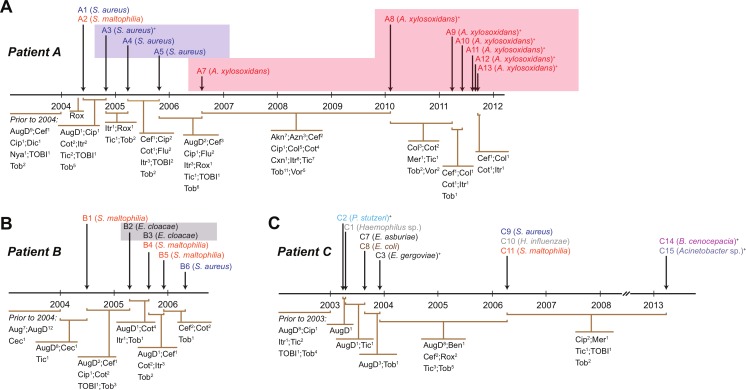
Timing of isolation of each sequenced strain for Patient A (A), Patient B (B) and Patient C (C). Antibiotic treatment administered between each strain isolation point is given below each timeline, with the number of courses denoted by superscript numbers. Shaded areas indicate strain persistence. +Samples obtained from sputum. Aug, amoxicillin/clavulanic acid (Augmentin); AugD, amoxicillin/clavulanic acid (Augmentin Duo); Akn, amikacin; Azn, azithromycin; Ben, benzylpenicillin sodium (BenPen); Cec, cefaclor monohydrate (Ceclor); Cef, ceftazidime; Cip, ciprofloxacin (Ciproxin); Col, colistin; Cxn, cefoxitin; Dic, dicloxacillin; Flu, flucloxacillin (Fluclox); Itr, itraconazole; Mer, meropenem; Nya, nystatin (Mycostatin); Rox, roxithromycin (Rulide); Sxt, trimethoprim/sulfamethoxazole (Bactrim); Tic, ticarcillin/clavulanic acid (Timentin); TOBI, nebulised tobramycin; Tob, Tobramycin; Vor, voriconazole.

**Figure 2:**
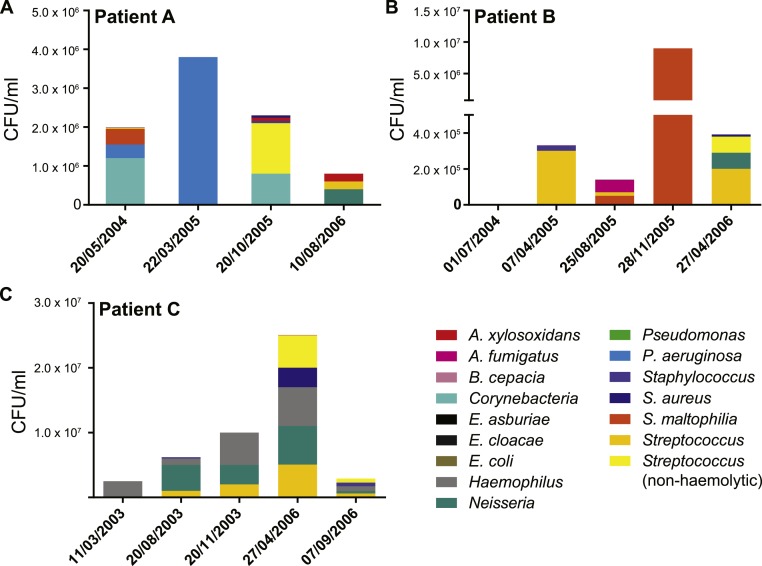
Patient specific profiles were determined from the culturable fraction of each patients’ lung microbiome. CFU/ml of culturable species contained in each BAL sample was calculated from plate counts at each time point. Details contained in Table S1.

We collected data from three children with cystic fibrosis (patients A, B and C; each under 3 years of age at start of study period) receiving treatment at The Royal Children’s Hospital, Brisbane, between 2003 and 2013. The culturable fraction of each patients’ lung microbiome was determined using bronchoalveolar lavage (BAL) plate counts and colony identifications revealing patient specific profiles (Figs. 1 and 2, Table S1). These isolate profiles also differed substantially between sampling dates in both patient A and patient B. The third patient, patient C, displayed a stable profile with increasing density. This kind of inter- and intrapatient variability of microbial communities amongst CF patients has been previously reported in both adults and children (Zhao et al., 2012; Hampton et al., 2014). We selected 28 isolates from both BAL and sputum samples for genome sequencing. Selection criteria included the availability of serial isolates of the same species to permit analysis of microevolution or multiple genera from the same patient for the purposes of investigating lateral gene transfer. Draft genomes of 28 isolates were assembled covering several key non-pseudomonal genera, including *Staphylococcus, Achromobacter* and *Stenotrophomonas* (Table 1). Estimations of the completeness and contamination of these genomes indicate that they are near complete (≥90%) with low contamination (≤5%; Parks et al., 2015).

**Table 1:** Details of CF isolates sequenced in this study. Bold strains are reference genomes used in analysis. Completeness and contamination measured at the species level where possible, otherwise at genus level where indicated (*).

Sample	Species	Isolation date	Sample source	CFU/ml	Scaffolds	Total length	Completeness	Contamination
***MSSA476***	***Staphylococcus aureus***	***1998***				***2,799,802***	***99.11%***	***0.39%***
A1	*S. aureus*	20/05/2004	BAL	9.0 × 10^3^	20	2,876,283	98.52%	0.41%
A3	*S. aureus*	21/10/2004	Sputum	–	21	2,752,347	99.09%	0.39%
A4	*S. aureus*	22/03/2005	BAL	2.8 × 10^4^	19	2,750,729	99.09%	0.39%
A5	*S. aureus*	20/10/2005	BAL	6.0 × 10^4^	19	2,751,323	99.09%	0.39%
B6	*S. aureus*	27/04/2006	BAL	1.0 × 10^4^	17	2,710,122	99.11%	0.39%
C9	*S. aureus*	27/04/2006	BAL	3.0 × 10^6^	16	2,724,382	98.93%	0.54%
***NH44784***	***Achromobacter xylosoxidans***	***1996***				***6,916,670***	***95.12%***	***6.40%***
A7	*A. xylosoxidans*	10/08/2006	BAL	2.0 × 10^5^	33	6,519,357	95.18%	4.27%
A8	*A. xylosoxidans*	21/02/2010	Sputum	–	29	6,414,199	94.96%	4.32%
A9	*A. xylosoxidans*	14/03/2011	Sputum	–	31	6,520,769	95.41%	4.16%
A10	*A. xylosoxidans*	30/05/2011	Sputum	–	36	6,518,157	95.18%	4.32%
A11	*A. xylosoxidans*	5/08/2011	Sputum	–	41	6,517,757	95.18%	4.27%
A12	*A. xylosoxidans*	18/08/2011	Sputum	–	29	6,519,737	95.41%	4.27%
A13	*A. xylosoxidans*	5/09/2011	Sputum	–	32	6,522,295	95.30%	4.27%
***K279a***	***Stenotrophomonas maltophilia***	***1998***				***4,851,126***	***99.85%***	***0.08%***
A2	*S. maltophilia*	20/05/2004	BAL	4.0 × 10^5^	14	4,528,500	99.49%	0.39%
B1	*S. maltophilia*	1/07/2004	BAL	3.9 × 10^3^	14	4,339,251	99.51%	0.24%
B4	*S. maltophilia*	25/08/2005	BAL	5.0 × 10^4^	13	4,346,482	95.28%	0.74%
B5	*S. maltophilia*	28/11/2005	BAL	9.0 × 10^6^	24	4,359,348	98.20%	1.73%
C11	*S. maltophilia*	27/04/2006	BAL	4.0 × 10^4^	52	4,798,863	98.42%	2.71%
B2	*Enterobacter cloacae*	7/04/2005	BAL	2.0 × 10^2^	52	4,984,846	95.89%	1.69%
B3	*E. cloacae*	25/08/2005	BAL	3.0 × 10^2^	53	5,002,539	95.89%	1.73%
C3	*Enterobacter gergoviae*	17/11/2003	Sputum	–	70	5,748,688	95.92%*	5.20%
C7	*Enterobacter asburiae*	20/08/2003	BAL	1.0 × 10^3^	45	4,941,126	99.98%*	0.05%
C8	*Escherichia coli*	20/08/2003	BAL	3.0 × 10^3^	52	5,094,694	98.99%	0.58%
C1	*Haemophilus* sp.	11/03/2003	BAL	2.5 × 10^6^	21	2,002,441	99.74%*	1.38%
C10	*Haemophilus influenzae*	27/04/2006	BAL	6.0 × 10^6^	19	1,875,554	99.73%	0.02%
C2	*Pseudomonas stutzeri*	3/03/2003	Sputum	–	18	4,347,030	99.57%	0.13%
C14	*Burkholderia cenocepacia*	6/03/2013	Sputum	–	28	7,276,147	96.65%	1.76%
C15	*Acinetobacter* sp.	7/03/2013	Sputum	–	53	3,549,490	99.96%*	0.60%

To determine whether the isolates obtained represent atypical examples of their species we considered both their antibiotic sensitivity and overall virulence gene complement. Antibiotic sensitivity testing showed a mixture of susceptible and resistant strains (Table 2). The *S. aureus* isolates were sensitive to most antibiotics tested, as is typical of methicillin-sensitive *S. aureus* (Nimmo et al., 2011), while naturally multi-drug resistant *A. xylosoxidans* isolates displayed characteristic resistance to multiple classes of compounds (reviewed in Conway et al., 2003). Resistance fluctuated between sequential *A. xylosoxidans* isolates, making this a particularly interesting series to examine further. Comparison of virulence genes for each species with the key CF disease causing agent *P. aeruginosa* showed *P. aeruginosa* substantially exceeded all species isolates in the categories of adherence and secretion and most species in host immune evasion (Table 3). This could be expected based on the success of *P. aeruginosa* in the CF lung, however may also be a product of database bias against less well characterised species. With no obvious trend emerging from this broad analysis we undertook analysis of the serially infecting species to look for more subtle changes occurring during infection.

**Table 2:** Antibiotic susceptibility testing of analysed isolates. Coloured squares indicate *in vitro* susceptibility testing results. Reference provides an indication of typical resistance of the species based on (*x*) papers: 0% resistance (−), <25% resistance (1), 25–50% resistance (2), 50–75% resistance (3), >75% resistance (4). *P. aeruginosa* isolate $\mathrm{A}\hat {}$ was not sequenced. BCC, *Burkholderia cepacia* complex; Pip + taz, piperacillin plus tazobactam; Tic + clav: ticarcillin plus clavulanic acid; Sxt: trimethoprim plus sulfamethoxazole. References used for averages contained in Table S3.

	Amikacin	Gentamicin	Tobramycin	Ampicillin	Aztreonam	Penicillin	Flucloxacillin	Pip + taz	Tic + clav	Meropenem	Ceftazidime	Ceftriaxone	Cephalothin	Cefotaxime	Vancomycin	Teicoplanin	Ciprofloxacin	Erythromycin	Clindamycin	Tetracycline	Sxt	Colistin	Rifampicin	Fusidic acid
***Pseudomonas aeruginosa***																								
*Reference (3)*	**2**	**3**	**2**		**1**			**2**	**2**	**2**	**2**			**4**			**2**			**4**	**4**	**1**		
$\mathrm{A}\hat {}$	s	s	s					s	s		s						s					s		
***Staphylococcus aureus***																								
*MSSA (2)*		**1**				**4**										**-**	**1**	**1**	**1**	**1**	**1**		**1**	**1**
*MRSA (2)*		**2**				**4**										**-**	**2**	**3**	**3**	**2**	**2**		**1**	**1**
A1		s				r	s						s		s	s	s	s	s	s	s		s	r
A3/4/5		s				r	s						s		s	s	s	s	s	s	s		s	s
C9						r	s						s		s	s	s	s	s		s		s	s
B6						r	s						s		s	s	s	s	s	s	s		s	s
***Achromobacter xylosoxidans***																								
*Reference (3)*	**2**	**3**	**3**		**4**			**2**	**1**	**2**	**1**			**2**			**2**			**3**	**1**	**1**		
A7		r	r	r					s	s		r	r				r				s			
A8		r	r	r	r				r	r		r	r				r				r	s		
A9		r	r	r	r			s	s	r			r				r				r	r		
A10		r	r	r					r	r		r	r				r				r	r		
A11		r	r	r	r				s	r	s		r				r					s		
A12		r	r	r	r				r	r	r		r				r					r		
A13		r	r	r	r				r	r	r		r				r					r		
***Stenotrophomonas maltophilia***																								
*Reference (2)*	**3**	**2**	**3**		**4**			**4**	**2**	**4**	**3**			**4**			**3**			**4**	**3**	**1**		
B1																					s			
B4																					s			
B5																					s			
A2																					s			
C11																					s			
***Haemophilus influenzae***																								
*Reference (3)*				**1**								**-**		**-**			**1**				**1**			
C1				s										s							s			
C10				s										s							s			
***Enterobacter***																								
*E. cloacae (3)*	**1**	**-**	**1**		**1**	**4**		**1**		**-**	**1**	**1**		**1**			**1**	**4**	**4**	**1**				
B2	s	s	s	r					s	s	s	s	r				s				s			
B3	s	s	s	r				s	s	s	s	s	r				s				r			
C3	s	s	s	r					r	s	r	r	r				s				s			
C7	s	s	s	r					r	s		r	r				s				s			
***Burkholderia cenocepacia***																								
*BCC (2)*	**4**	**4**	**4**		**3**			**2**	**4**	**2**	**2**						**4**				**4**			
C14	r	r	r	r					r	s	s						s				s	r		
***Acinetobacter baumannii***																								
*Reference (2)*	**1**	**3**						**2**	**2**	**1**	**2**						**3**				**2**			
C15	s	r	r					s	s	s	s						s				s	s		
***Escherichia coli***																								
*Reference (2)*		**1**		**2**							**1**	**1**	**1**				**1**			**2**	**1**			
C8	s	s	s	r					r	s			r	s			s				s			
***Pseudomonas stutzeri***																								
C2	s	s	s						s	s	s						s							

**Table 3:** Virulence factor gene distribution between sequenced species in comparison with *P. aeruginosa.* Table constructed from number of hits in VFDB with over 60% coverage of target and over 80% identity. *Haemophilus* and *Enterobacter* are an average of the isolates obtained for each genus.

	*P. aeruginosa*	*S. aureus*	*S. maltophilia*	*A. xylosoxidans*	*Haemophilus*	*Enterobacter*	*E. coli*	*B. cenocepacia*	*Acinetobacter*	*P. stutzeri*
Adherence	**72**	**16**	**25**	**25**	**5**	**38**	**33**	**51**	**2**	**53**
Motility	**3**		**4**	**5**		**5**	**2**	**8**		**1**
Host immune evasion	**19**	**19**	**5**	**3**	**1**	**2**		**13**	**1**	**7**
Iron uptake	**17**	**1**		**2**	**10**	**27**	**45**	**10**		
Lipase	**1**	**1**								
Protease	**3**	**4**			**1**	**1**			**1**	
Exoenzyme		**4**								
Quorum sensing systems	**3**							**1**		
Regulation	**5**			**1**		**8**	**6**	**3**	**1**	**1**
Type II SS						**1**	**11**			
Type III SS	**39**			**14**				**4**		
Type VI SS	**5**			**1**						
Type VII SS		**8**								
Toxin	**1**	**13**				**1**				
Endotoxin					**5**	**2**	**1**			
Uncategorised	**35**	**8**	**10**	**16**	**16**	**54**	**69**	**13**	**9**	**25**

### *Staphylococcus aureus*

**Figure 3:**
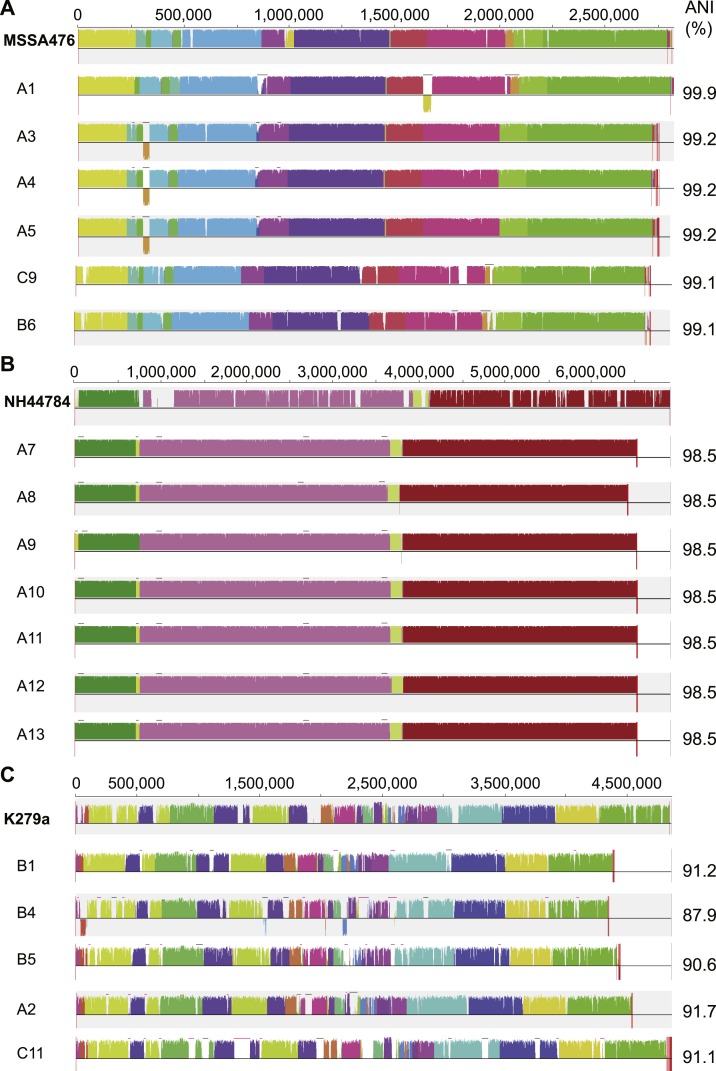
Similarity of *S. aureus* (A), *A. xylosoxidans* (B) and *S. maltophilia* (C) isolates with reference strains used for analysis. Each draft genome was aligned to the chosen reference strain for each species. Phage regions marked with black bars, genomic islands marked with pink bars. ANI calculated in comparison to each reference strain.

*S. aureus* is a commensal organism commonly found inhabiting the nasal passage (Wertheim et al., 2005). When disease occurs, it is usually caused by the patient’s own colonising strain (Von Eiff et al., 2001). In CF patients, *S. aureus* can contribute to inflammation (Sagel et al., 2009) and to the development of pneumonia (Koulenti et al., 2009). We sequenced six *S. aureus* isolates; four were obtained from patient A and a single isolate was obtained from both patient B and C (Fig. 1). Draft assemblies were of similar size with ∼170 kb (6%) separating the smallest, B6, from the largest, A1 (Table 1). Multilocus sequence type analysis classified A1 as sequence type ST1, A3, 4 and 5 as ST15, C9 as ST6 and B6 as ST953 (Enright et al., 2000). Contigs were ordered against MSSA476 (NCBI acc. no. BX571857), a community acquired strain isolated in 1998 from an immunocompetent child with a severe invasive infection (tibial osteomyelitis) and bacteraemia (Holden et al., 2004). The patient A strains A3–A5 appeared substantially different from the earliest isolated strain A1 while being very similar to each other suggesting the persistence of a single strain for a year (Figs. 1 and 3A). *S. aureus* infections are typically persistent, with over 80% of patients colonised by the same lineage for over two years (Branger, Gardye & Lambert-Zechovsky, 1996; Vu-Thien et al., 2010). SNP analysis within the patient A strains further confirmed the close relationship between A3, 4 and 5; A4 and A5 share one SNP that causes a synonymous change and A5 contains two SNPS, both of which are intergenic. Between A1 and A3, 4 and 5, large differences are associated with prophage regions and genomic islands (Fig. 3A).

#### The analysed *S. aureus* strains do not contain consistent virulence factor profiles

*S. aureus* can utilise a range of virulence factors to promote infection including secreted toxins, immune evasion and various host interactions (Gordon, 1998; Cheung et al., 2004). We examined each strain for virulence factor encoding genes in comparison with four previously characterised reference strains including MSSA476 (Table S2). We found A1 to contain more enterotoxin genes than the other strains, with the same profile as the reference strains MSSA476 and MW2. The similarity of MSSA476 and MW2 has been noted previously (Holden et al., 2004). In other virulence factor categories patterns were less obvious. Within patient A, A1 contains staphylokinase and collagen binding protein not found in A3, 4 and 5. Evidence supports staphylokinase as having a role both in invasiveness but also in symbiosis (Bokarewa, Jin & Tarkowski, 2006) while collagen binding protein is found more commonly in invasive isolates (Peacock et al., 2002). Both C9 and B6 contain staphylokinase while only C9 contains collagen binding protein. A3, 4 and 5 contain chemotaxis inhibitory protein capable of inhibiting C5a-induced recruitment of neutrophils (De Haas et al., 2004). They are the only strains to contain this gene. A1 contains a deletion within its capsule locus that truncates one of the genes necessary for capsule production, *capH.* While acapsular strains are more adherent to host cells (Pöhlmann-Dietze et al., 2000) they are also more prone to phagocytosis (Karakawa et al., 1988), thus the capsule may be a benefit or a hindrance at different stages of infection. Where capsule change is observed over an infection time course, later stage isolates are more likely to have increased capsule size than decreased (Hirschhausen et al., 2013). Being potentially unable to produce capsule when required may have limited A1’s ability to infect long-term, as suggested by its isolation at a single time point. However, as only a single isolate was sequenced at each time point, it is possible A1 persisted undetected.

#### Antibiotic resistance is limited in all analysed *S. aureus* isolates

Antibiotic sensitivity testing revealed that the six *S. aureus* isolates analysed in the present study were sensitive to a wide range of antibiotics (Table 2). Their broad sensitivity is typical of methicillin-sensitive *S. aureus* while methicillin-resistant strains more commonly exhibit resistance to multiple antibiotic classes (Nimmo et al., 2011) (Table 2). A1 was also resistant to fusidic acid while all other strains were sensitive. This pattern was supported genomically with all strains harbouring the *β*-lactam resistance genes *blaZ*/*blaI*/*blaRI* encoded on a plasmid (Table S2 and Fig. S1); a second chromosomal copy of the gene trio was also found in A1. The fusidic acid resistance gene, *fusC*, was identified in A1 within a cassette insertion at the staphylococcal chromosome cassette SCC*mec* attachment site (reviewed in Hanssen & Sollid, 2006). This same insertion is found in the reference strain MSSA476 (Holden et al., 2004). The fosfomycin resistance gene *fosB* was found in A3, 4, 5 and C9. However, C9 contains a single base deletion within the gene creating a frameshift at residue 36 of 139. Fosfomycin is rarely used and was not administered to these patients. We also checked the sequence of particular genes known to contain mutations conferring drug resistance in other strains, including *rpoB*, *gyrA/B*, *parC/E*, and *fmtC*, however while there were some differences in sequence, none contained mutations at sites previously characterised and known to confer resistance. Thus overall we found no evidence to contradict the reported susceptibility of the analysed *S. aureus* strains that would make them difficult to treat.

#### *S. aureus* population persistence does not correlate with antibiotic administration

Given the sensitivity of the *S. aureus* strains to most of the tested antibiotics, we were surprised to see the persistence of the species in patient A and specifically the increasing population of A3, 4 and 5 seen for nearly a year (Table S1). Ciprofloxacin was administered both prior to the isolation of A1 from BAL fluid and subsequently prior to the isolation of A3 from sputum (Table S4). Susceptibility testing indicated both strains should have been sensitive to this drug. In addition, the combination drugs ticarcillin disodium plus clavulanate potassium (Timentin, IV administration) and amoxicillin plus clavulanic acid (Augmentin Duo, oral administration) were given and would be expected to have activity against these strains. Between the isolation of A3 and A5 the population steadily increased (Table S1) despite continued administration of Timentin as well as ciprofloxacin, sulfamethoxazole plus trimethoprim and flucloxacillin which were effective *in vitro* (Table 2). After the isolation of A5, amoxicillin plus clavulanic acid was administered to the patient which was the only drug that had not been given to the patient since before the isolation of A3. No *S. aureus* was reported at the following time point, however this may have been associated with the general decrease in bacterial load observed at this time (Fig. 2A) rather than a product of specific antibiotic treatment.

A potential source of resistance for this apparently susceptible population could have been other species present over the same period. There was a large *Pseudomonas* population present at the time point at which A4 was isolated. Aminoglycoside and macrolide modifying enzymes can be found in *P. aeruginosa* and if secreted in a fashion similar to *β*-lactamases (Ciofu et al., 2000) may be a plausible explanation for the accumulation of *S. aureus* between the time points of A3 and A5 in the presence of these drugs. However, susceptibility testing of a *P. aeruginosa* strain isolated at the same time as A4 (Table S1) revealed broad sensitivity in this isolate (Table 2). Thus the pattern of presence despite relevant antibiotic treatment extends to this population as well suggesting that sufficient antibiotics are not reaching sensitive *in situ* populations or potentially issues with patient compliance. The formation of biofilms by infecting species is a likely contributor to this inconsistency due to their ability to interfere with the activity of some antibiotics (reviewed in Fux et al., 2005), a situation that can be exacerbated when multiple species are present, requiring a much higher dose for eradication (Lopes et al., 2012). These results call into question the usefulness of standard antibiotic susceptibility testing in the design of patient treatment regimes and are consistent with multiple studies reporting little or no correlation between *in vitro* testing and patient outcome (reviewed in Doern & Brecher, 2011; Doering et al., 2012).

### *Achromobacter xylosoxidans*

*A. xylosoxidans* is an opportunistic, multi-drug resistant organism found in a variety of aqueous environments including water distribution systems (Amoureux et al., 2013a). While its role in CF is still under investigation, *A. xylosoxidans* has been shown to induce inflammation in these patients (Hansen et al., 2010). Chronic infection is also associated with declining lung function in some cases (Rønne Hansen et al., 2006). We sequenced seven *A. xylosoxidans* isolates, all from patient A. The initial strain originated from a BAL sample while all subsequent isolates were obtained from sputum samples. Assembly revealed a similar genome size for all strains except A8 which was ∼100 kb smaller than the other strains (Table 1). Contigs were ordered against NH44784-1996 (NCBI acc. no. HE798385), an isolate obtained from a CF patient in 1996 (Jakobsen et al., 2013). All *A. xylosoxidans* strains had a high degree of synteny (Fig. 3B) suggesting that they represent a single population that persisted from 2006 to 2011 (Fig. 1). This was confirmed via SNP analysis that revealed only a small number of unique mutations in all isolates except A7 (Table S5). A8–A13 also share five SNPs and A9–A13 share 17 SNPs and 3 indels. A11 contains considerably more mutations than the other strains which points to the possibility of an increased mutation rate in this isolate. Non-synonymous SNPs were found in three DNA repair associated proteins: RadC (A>G-Asp146Gly), MutL (C>T-Pro457Ser) and MutS (A>C-Thr810Pro). The transition:transversion ratio (∼10:1) supports a defect in MutS contributing to this mutation signature (Table S5) (Young & Ornston, 2001).

#### Antibiotic resistance fluctuates within *A. xylosoxidans* isolates despite their close relationship

*A. xylosoxidans* exhibits natural resistance to a wide range of antibiotics including aminoglycosides, cephalosporins (except ceftazidime) and the *β*-lactam aztreonam (Glupczynski et al., 1988; Turton et al., 2011; Amoureux et al., 2013b) (Table 2). Two RND-type multidrug efflux pump systems have been shown to contribute to this resistant phenotype (Bador et al., 2011; Bador et al., 2013). The first, AxyABM, is active against cephalosporins (except cefepime), aztreonam, nalidixic acid, fluoroquinolones, and chloramphenicol to varying degrees (Bador et al., 2011). The second, AxyXY-OprZ provides significant resistance to multiple aminoglycosides and is also capable of removing cefepime, carbapenems, some fluoroquinolones, tetracycline, and erythromycin (Bador et al., 2013). In addition to the characterised AxyXY-OprZ and AxyABM pumps, *A. xylosoxidans* contains a variety of putative resistance genes, potentially up to 50 genes in total (Hu et al., 2015). All patient A strains contained the genes for the AxyABM and AxyXY-OprZ pumps and displayed resistance accordingly to aminoglycosides, cephalosporins, ciprofloxacin and aztreonam (Table 2). Sequence identity across both pump systems was 100% except for a single non-synonymous SNP (Ile625Ser) found in AxyB of A8 that does not appear to affect its *in vitro* resistance.

Resistance to ticarcillin plus clavulanic acid, meropenem, ceftazidime, sulfamethoxazole plus trimethoprim and colistin varied amongst the isolates during the time period (Table 2). Resistance to meropenem and sulfamethoxazole plus trimethoprim appeared in A8 and was seen in all subsequent tested isolates. Sulfamethoxazole/trimethoprim had not been administered to the patient for over a year prior to the isolation of the sensitive A7, but was given two weeks following and between the isolation of A8 and A9 and A9 and A10. Thus, antibiotic therapy may have contributed to the maintenance of resistance to this drug. Meropenem was administered only once during the study period, one year after resistance was documented in A8, thus in this case resistance could not have been a direct consequence of therapy. Genome analysis revealed several possible contributors to the emergence of resistance to these two drugs. Firstly, A8 to A13 share common mutations within two transcriptional regulators; the first of the AraC family (Ile77Asn, NH44784 locus NH44784_036441) and the second of the TetR family (Ala154Val, NH44784 locus NH44784_061441). The sequence of A7 at these positions matches the reference strain NH44784-1996, also sensitive to meropenem and sulfamethoxazole/trimethoprim. Another possible explanation for the observed change in resistance is a small deletion (330 nt) in an additional AraC regulator (NH44784 locus NH44787_013001) in A8 to A13 that is absent in both A7 and NH44784-1996.

The greatest fluctuation in sensitivity was observed to ticarcillin plus clavulanic acid, with sensitivity re-emerging in A9 and the putative mutator strain A11 after resistance developed in A8 (Table 2). While this drug was administered following the isolation of A7, the last recorded course for the patient was given 6 months prior to the isolation of A8, therefore this cycling of resistance does not reflect cycling of antibiotic therapy. Resistance appearing in A8 and then being lost due to mutation in A9 and A11 would be the simplest explanation for this fluctuation and there were no mutations in A8, 10, 12 and 13 that were likely to have led to resistance appearing in these strains independently. By contrast, A9 and A11 both contain SNPs within an ABC transporter (NH44784 locus NH44784_038441) creating the same non-synonymous mutation at two different locations: Leu388Pro in A9 and Leu446Pro in A11. Resistance to colistin also fluctuated during the period (Table 2) and also does not appear obviously connected to antibiotic therapy as the drug was administered two months prior to the isolation of both the sensitive A8 and the resistant A9. Therefore in *A. xylosoxidans*, as in *S. aureus*, there is disagreement between antibiotic sensitivity testing and population persistence.

### *Stenotrophomonas maltophilia*

*S. maltophilia* is an opportunistic, multi-drug resistant organism that is found in soil and aqueous environments also including water distribution systems (reviewed in Brooke, 2012). The incidence of *S. maltophilia* in CF patients varies (Goss et al., 2004; Dalbøge et al., 2011) but has been shown to be increasing in some locations (Emerson et al., 2010). Analyses of the impact of *S. maltophilia* infection are contradictory with some studies reporting little or no effect on lung function (Goss et al., 2004; Dalbøge et al., 2011) while others report poorer clinical status (Talmaciu et al., 2000) and an increased risk of pulmonary exacerbation with chronic infection (Waters et al., 2011). We sequenced five *S. maltophilia* isolates, three from patient B, one from patient A and one from patient C. Contigs were ordered against the reference strain K279a (NCBI acc. no. AM743169), isolated in 1998 from a bloodstream infection (Crossman et al., 2008). *S. maltophilia* strains were relatively divergent from each other compared to the *S. aureus* and *A. xylosoxidans* strains, even those from patient B (Fig. 3C) which is typical of *S. maltophilia* infection, particularly in young patients (Valdezate et al., 2001; Pompilio et al., 2011). Notably, average nucleotide identity (ANI) analysis revealed all strains lacked sufficient identity to the reference strain to be classified as the same species (<95%; Fig. 3C; Goris et al., 2007) despite forming a robustly monophyletic clade (Fig. S2). Comparison between available reference genomes also produced low identity e.g., 91.5% ANI between K279a and JV3 (NCBI acc. no. NC_015947). Based on this type of analysis, it has been suggested that strains currently designated as *S. maltophilia* actually represent a complex (Svensson-Stadler, Mihaylova & Moore, 2012) placing a caveat on interstrain comparisons.

*S. maltophilia* is intrinsically multi-drug resistant with resistance reported against multiple classes of antibiotics (reviewed in Brooke, 2012). A substantial degree of resistance is conferred by the presence of a number of efflux pump systems. The reference strain K279a has nine efflux pumps of which SmeZ confers resistance to aminoglycosides and SmeJ/K contributes minor aminoglycoside, fluoroquinolone and tetracycline resistance (Crossman et al., 2008). All nine are present within each of the analysed *S. maltophilia* isolates (Table S6). Other pumps are typically associated with resistance following mutations leading to over-expression; consequently their presence alone does not indicate resistance (reviewed in Brooke, 2012). We also examined the presence of other putative and known resistance genes annotated in the K279a genome (Crossman et al., 2008) (Table S6). All except two were found in at least one of the analysed isolates but with varying identity. This variation is known to contribute to differing degrees of antibiotic resistance (Avison et al., 2001) and further complicates resistance predictions based on genome data.

All *S. maltophilia* isolates were susceptible to sulfamethoxazole plus trimethoprim, the preferred treatment option for this species but against which resistance is becoming an increasing problem (Brooke, 2012) (Table 2). Patient B received sulfamethoxazole plus trimethoprim throughout the study period. Given the dissimilarity of the patient B strains it is possible the patient is being sequentially colonised by this species rather than there being a persistent population, suggesting in this regard antibiotic treatment was effective. However, it is also possible that there exists a diverse infecting population in this patient with a different lineage sampled at each time point. Patient A also received sulfamethoxazole plus trimethoprim after the isolation of A2 and no *S. maltophilia* was noted after this time. Like patient A, a single *S. maltophilia* isolate was obtained from patient C, however no sulfamethoxazole plus trimethoprim was administered in this case. Ticarcillin plus clavulanic acid was given following the isolation of C11 and may have cleared the infection in this patient.

### Other CF isolates

Beyond the three main species described above, several other species were obtained that appeared sporadically in the patient profiles. Two *Enterobacter cloacae* strains were isolated, both from patient B. The genomes of the two *E. cloacae* isolates are very similar indicating persistence; we found only a single, intergenic SNP separating the two, however both contain unique sections associated with mobile elements (Fig. S3A). One of these regions in B3 is similar to antibiotic resistance elements from multiple species including *E. coli* (Tn2610, NCBI acc. AB207867; Tn21, NCBI acc. AF071413) and *A. baumannii*, (AbaR21, NCBI acc. KM921776) but with a different arrangement and contains an antiseptic resistance gene QacE delta 1, a sulfonamide insensitive dihydropteroate synthase Sul1 and a trimethoprim insensitive dihydrofolate reductase DfrA5. B3 was resistant to sulfamethoxazole plus trimethoprim supporting the activity of this element in the isolate (Table 2). Sulfamethoxazole plus trimethoprim had not been given to the patient for eight months prior to the isolation of B2 but was administered four times over the four month period separating B2 and B3, potentially providing the selective pressure for insertion of this element (Table S4). In addition, the similarity of the two *E. cloacae* isolates supports a lateral acquisition by the later isolate during infection. There was no similarity between the element and the other sequenced isolates from patient B and no BLAST hits were returned from *Staphylococcus* or *Streptococcus* species, which were also present in BAL samples during this period, indicating an unsampled source for this element. High sequence identity of the laterally transferred gene cassette with other members of the family Enterobacteriaceae (>95% identity), albeit with variable gene order (Fig. S4), suggest that this lineage likely harbours the donor species.

We also obtained two *Haemophilus* isolates, both from patient C. Neither strain contained the capsule biosynthesis locus and thus represent nontypeable *H. influenzae* (NTHi) which make up the majority of respiratory mucosal associated infections (Erwin et al., 2008). NTHi are a diverse population (Erwin et al., 2008), reflecting their ability to be both commensal and pathogenic, and this diversity is illustrated in the two analysed strains which were obviously dissimilar (Fig. S3B). Despite this dissimilarity, both strains displayed susceptibility to all three antibiotics tested: ampicillin, cefotaxime and sulfamethoxazole plus trimethoprim. Differences were observed, however, between the virulence factor genes present in the two strains. C10 contains the high-molecular weight adhesins HMW1 and HMW2 which are the major adhesins in ∼80% of NTHi strains (St. Geme et al., 1998) and are associated with acute otitis media (Krasan et al., 1999; Ecevit et al., 2004). Strains lacking HMW1 and HMW2 typically contain an alternative adhesin, *hia* (St. Geme et al., 1998), however C1 does not contain this gene either. C1 does however contain a haemagglutinating pili gene cluster consisting of five genes, absent from C10. These clusters are predicted to be important for nasopharyngeal colonisation (Krasan et al., 1999; Ecevit et al., 2004). C1 also contains a unique Type I CRISPR-Cas system (Makarova et al., 2011) resembling that of *H. haemolyticus* strain M21621 (NCBI acc. NZ_AFQQ01000000). Four spacer sequences were contained within the region. No CRISPR system was identified in C10. The divergent nature of these two isolates obtained from the same patient is typical of NTHi where sequential isolates are generally found to be different strains (Kaur et al., 2011; Cardines et al., 2012). Moreover, ANI and concatenated marker gene analysis of the genomes suggests that isolate C1 is not an *H. influenzae* strain (<95% ANI, Fig. S3B; Goris et al., 2007) having a closer ANI to *H. haemolyticus* (ANI 93.8 vs. 91.9%) and clustering with representatives of this species (Fig. S5).

### Absence of lateral acquisition of antibiotic resistance genes and virulence factors between investigated species

The CF lung represents a diverse microbial community with sputum and BAL samples typically yielding multiple species per sample (Harris et al., 2007; Bittar et al., 2008). This cohabitation provides scope for lateral gene transfer both within and between species and the selective pressure applied by continual antibiotic therapy, coupled with the potential for antimicrobials to induce phage movement, means the CF microbial community needs to be considered as a whole (reviewed in Rolain et al., 2011). We were unable to identify any potential examples of lateral transfer occurring between isolates within the analysed dataset that could not be explained by shared evolutionary history. This may be due to the temporal isolation of most of the isolates, with few being obtained at the same time point. It is also likely a product of our relatively small sample size and the study of a single isolate at each time point. The *E. cloacae* example discussed above supports the possibility of identifying transfer events from outside the dataset, therefore we also examined predicted mobile elements in each of the strains for evidence of general acquisition. While we identified some elements with unique arrangements, we found no further evidence of recently acquired elements containing antibiotic resistance genes.

## Conclusions

Here we have used whole-genome sequencing to analyse a series of non-pseudomonal CF isolates looking for indicators of the emergence of antibiotic resistance or unique virulence traits in this group. We found nothing to suggest that this collection of isolates would exhibit altered virulence in comparison with other isolates of the same species. This is in agreement with a number of studies that have demonstrated virulence factors required for acute infection are less important in chronic infection (Smith et al., 2006; Rau et al., 2010; Nakamura et al., 2011). It also speaks to the nature of CF where the barriers to infection are lowered. We do, however, acknowledge that as an entirely genome based analysis this study could not detect subtle differences (for example, regulatory changes) that may be present in these isolates. In addition, we do not have data related to the overall condition of each patient during the study period so we are unable to correlate the presence of these isolates with episodes of exacerbation or general lung function. We showed that *S. aureus* and *A. xylosoxidans* are able to persist despite appropriate antibiotic therapy, implying insufficient antibiotic reaching susceptible populations through physical barriers such as e.g., biofilms and/or inactivation of antibiotics by other community members. We identified only a single example of acquisition of antibiotic resistance by lateral transfer in *E. cloacae* from a closely related donor (same family) but did not see a general trend of association of antibiotic resistance or virulence components with mobile elements. We speculate that this may be due to lack of selective pressure by antibiotic therapy. The environmental multi-drug resistant *A. xylosoxidans* persisted for nearly five years with microevolution occurring through small mutations contributing to fluctuating antibiotic resistance. In contrast, we did not detect any evidence of persistence of *S. maltophilia*, also naturally multi-drug resistant, although as we did not complete population level analysis this remains speculative. While limited by the analysis of a single isolate at each time point, this study adds to the available information regarding the characteristics of non-pseudomonal species in CF patients and highlights *A. xylosoxidans* in particular as a species of concern due to its ability to persist for long periods, which may or may not be associated with its antibiotic resistance.

## Supplemental Information

10.7717/peerj.1223/supp-1 Table S1 Species identified in BAL and sputum samples

10.7717/peerj.1223/supp-2 Table S2 Virulence factors identified in *S. aureus* isolates

10.7717/peerj.1223/supp-3 Table S3 Antibioitic resistance levels reported in the literature

10.7717/peerj.1223/supp-4 Table S4 Antibiotic treatment received by each patient during study period

10.7717/peerj.1223/supp-5 Table S5 SNPs identified in *A. xylosoxidans* isolates

10.7717/peerj.1223/supp-6 Table S6 Resistance genes identified in *S. maltophilia* strains based on reference stran K279a

10.7717/peerj.1223/supp-7 Figure S1 *β*-lactam antibiotic resistance is carried on a plasmid in all analysed *S. aureus* isolates progressiveMauve (Darling, Mau & Perna, 2010) alignment of plasmid sequences from each isolate in comparison with the MSSA476 plasmid pSAS1. Similarity of sequence is indicated by coloured sections. *β*-lactamase genes are indicated in red, cadmium resistance genes are indicated in green.

10.7717/peerj.1223/supp-8 Figure S2 Phylogenetic tree of the analysed *S. maltophilia* CF isolates in association with reference strains A maximum likelihood tree constructed using MEGA (Tamura et al., 2013) based on the concatenated alignment of 83 single copy genes generated using CheckM (Parks et al., 2015). The tree is drawn to scale, with branch lengths measured in the number of substitutions per site. Bootstrap (500 replicates) support values above 75% are shown.

10.7717/peerj.1223/supp-9 Figure S3 Similarity of *E. cloacae* (A) and *Haemophilus* (B) CF isolates with reference strains progressiveMauve (Darling, Mau & Perna, 2010) alignment of each strain with reference strain used for analysis. ANI calculated in comparison to the chosen reference strain.

10.7717/peerj.1223/supp-10 Figure S4 Antibiotic resistance cassette found in strain B3 shares high identity with other elements but contains a different arrangement Alignment generated using Easyfig (Sullivan et al., 2011).

10.7717/peerj.1223/supp-11 Figure S5 Phylogenetic tree of the analysed *Haemophilus* CF isolates in association with reference strains A maximum likelihood tree constructed using MEGA (Tamura et al., 2013) based on the concatenated alignment of 83 single copy genes generated using CheckM (Parks et al., 2015). The tree is drawn to scale, with branch lengths measured in the number of substitutions per site. Bootstrap (500 replicates) support values above 75% are shown.

## Additional Information and Declarations

### DNA Deposition

The following information was supplied regarding the deposition of DNA sequences: The draft assemblies from this project have been deposited at GenBank under the accessions LDVC00000000–LDWD00000000.
