# Supplementary material for: Comparative genomics of non-pseudomonal bacterial species colonising paediatric cystic fibrosis patients
Source: PeerJ. 2015 Sep 15;3:e1223. doi: 10.7717/peerj.1223 (PMC4579023; doi:10.7717/peerj.1223)
Supplement: Table S2 [file peerj-03-1223-s002.docx]

|  | | **Percent identity with reference gene (**⚫) | | | | | | | |
| --- | --- | --- | --- | --- | --- | --- | --- | --- | --- |
|  | **Locus** | **A1** | **A3/4/5** | **B6** | **C9** | ***MSSA476*** | ***MW2*** | ***MRSA252*** | ***N315*** |
| **Adherence** | | | | | | | | | |
| Autolysin | SAS0988 | 99% | 99% | 99% | 99% | ⚫ | 99% | 97% | 98% |
| Cell wall associated fibronectin binding protein | SAS1377 | 99% | 98% | 96% | 98% | ⚫ | 99% | 94% | 97% |
| Clumping factor A | SAS0752 | 98% | 90% | 89% | 90% | ⚫ | 98% | 90% | 88% |
| Clumping factor B | SAS2516 | 99% | 98% | 92% | 92% | ⚫ | 99% | 89% | 91% |
| Collagen adhesion | SAS2578 | 99% | - | - | 99% | ⚫ | 99% | 98% | - |
| Elastin binding protein | SAS1421 | 99% | 98% | 99% | 99% | ⚫ | 100% | 99% | 99% |
| Extracellular adherence protein | SAR2030 | 85% | 85% | 80% | 80% | 84% | 84% | ⚫ | 75% |
| Fibrinogen binding protein | SAS1091 | 100% | 99% | 99% | 99% | ⚫ | 100% | 93% | 99% |
| Fibronectin binding protein | SAS2388 | 100% | 87% | 88% | 88% | ⚫ | 100% | 86% | 94% |
| Fibronectin binding protein | SAS2387 | 99% | 86% | 86% | 85% | ⚫ | 98% | - | 87% |
| Intercellular adhesin *icaR/A/D/B/C* | SAS2551-5 | 100% | 99% | 99% | 99% | ⚫ | 100% | 99% | 99% |
| Ser-Asp rich fibrinogen-binding proteins *sdrC/D/E* | SAS0519-21 | 99% | 93% | 94% | 93% | ⚫ | 99% | 86% | 94% |
| Staphylococcal protein A | SAS0085 | 98% | 94% | 96% | 93% | ⚫ | 99% | 94% | 88% |
| Exported protein involved in expression of fibrinogen binding protein | SAS1092 | 100% | 86% | 86% | 100% | ⚫ | 100% | 93% | 100% |
| **Exoenzymes** | | | | | | | | | |
| Cysteine protease *sspB/C* | SAS0983-2 | 100% | 100% | 99% | 100% | ⚫ | 100% | 98% | 99% |
| Hyaluronate lyase | SAS2103 | 100% | 87%  91% | 92% 87% | 92% | ⚫ | 100% | 93%  81% | 95% |
| Lipase *lip* | SAS2556 | 99% | 99% | 99% | 99% | ⚫ | 100% | 98% | 99% |
| Lipase *geh* | SAS0297 | 100% | 99% | 99% | 99% | ⚫ | 100% | 98% | 99% |
| Serine V8 protease | SAS0984 | 99% | 96% | 98% | 95% | ⚫ | 98% | 94% | 97% |
| Staphylocoagulase | SAS0206 | 100% | 82% | 80% | 79% | ⚫ | 99% | 80% | 88% |
| Staphylokinase | SAS1868 | 100% | - | 99% | 100% | ⚫ | 99% | 99% | 99% |
| Thermonuclease | SAS0756 | 100% | 99% | 99% | 99% | ⚫ | 100% | 99% | 99% |
| **Host immune evasion** |  |  |  |  |  |  |  |  |  |
| *CapABCDEFG* | SAS0124-30 | ⭘ | 99% | 99% | 99% | ⚫ | 99% | 98% | 99% |
| *CapHIJK* (define capsule type) | SAS0131-4 | 8 | 8 | 5 | 8 | 8 | 8 | 8 | 5 |
| *CapLMNOP* | SAS0135-9 | 100% | 99% | 99% | 99% | ⚫ | 99% | 99% | 99% |
| Staphylococcal complement inhibitor | SAS1866 | 100% | 99% | 99% | 100% | ⚫ | 100% | 99% | 99% |
| Chemotaxis inhibitory protein | SAR2036 | - | 99% | - | - | - | - | ⚫ | 99% |
| Aureolysin | SAS2523 | 100% | 99% | 99% | 99% | ⚫ | 100% | 89% | 99% |
| **Secretion systems** | | | | | | | | | |
| Type VII secretion system *esxA* | SAS0258 | 100% | 100% | 100% | 100% | ⚫ | 100% | 99% | 100% |
| Type VII secretion system *esaA* | SAS0259 | 99% | 99% | 99% | 99% | ⚫ | 100% | 99% | 99% |
| Type VII secretion system *essA* | SAS0260 | 100% | 98% | 100% | 100% | ⚫ | 100% | 99% | 98% |
| Type VII secretion system *esaB* | SAS0261 | 100% | 99% | 93% | 99% | ⚫ | 100% | 98% | 99% |
| Type VII secretion system *essB* | SAS0262 | 100% | 99% | 96% | 98% | ⚫ | 100% | 99% | 99% |
| Type VII secretion system *essC* | SAS0263 | 100% | 94% | 98% | 99% | ⚫ | 100% | 96% | 99% |
| Type VII secretion system *esaC* | SAS0264 | 99% | - | 99% | 100% | ⚫ | 100% | - | 98% |
| Type VII secretion system *esxB* | SAS0265 | 100% | - | 99% | 100% | ⚫ | 100% | - | 97% |
| **Toxins** | | | | | | | | | |
| Alpha haemolysin | SAS1097 | 99% | 99% | 100% | 99% | ⚫ | 100% | 95% | 99% |
| Delta haemolysin | SAS1940a | 100% | 99% | 99% | 99% | ⚫ | 100% | 99% | 99% |
| Enterotoxin A | SAS1872 | 100% | - | - | 100% | ⚫ | 100% | 99% | 85% |
| Enterotoxin C | SA1817 | - | - | - | - | - | 98% | - | ⚫ |
| Enterotoxin G | SAR1916 | - | - | - | - | - | - | ⚫ | 98% |
| Enterotoxin H | SAS0051 | 100% | - | - | - | ⚫ | 100% | - | - |
| Enterotoxin I | SAR1919 | - | - | - | - | - | - | ⚫ | 97% |
| Enterotoxin-like K | SAS1921 | 100% | - | - | - | ⚫ | 100% | - | - |
| Enterotoxin-like L | SA1816 | - | - | - | - | - | ⚫ | - | - |
| Enterotoxin-like M | SAR1920 | - | - | - | - | - | - | ⚫ | 93% |
| Enterotoxin-like N | SAR1917 | - | - | - | - | - | - | ⚫ | 97% |
| Enterotoxin-like O | SAR1921 | - | - | - | - | - | - | ⚫ | 92% |
| Enterotoxin-like P | SA1761 | - | - | - | - | - | - | - | ⚫ |
| Enterotoxin-like Q | SAS1920 | 100% | - | - | - | ⚫ | 100% | - | - |
| Enterotoxin-like U selu | SAR1918 | - | - | - | - | - | - | ⚫ | - |
| Enterotoxin family protein | SAS1538 | 100% | 99% | 95% | 97% | ⚫ | 100% | 95% | 97% |
| Exfoliative toxin type A eta | SAS1106 | 100% | 99% | 99% | 99% | ⚫ | 100% | 97% | 99% |
| Exotoxin set1 | SAR0428 | - | - | - | - | - | - | ⚫ | - |
| Exotoxin set2 | SAR0425 | - | - | - | - | - | - | ⚫ | - |
| Exotoxin set3 | SAR0427 | - | - | - | - | - | - | ⚫ | - |
| Exotoxin set4 | SAR0431 | - | - | - | - | - | - | ⚫ | - |
| Exotoxin set5 | SAR0429 | - | - | - | - | - | - | ⚫ | - |
| Exotoxin set16 to 26 | SAS0384-96 | 99% | 95% | 94% | 95% | ⚫ | 99% | - | 95% |
| Haemolysin gamma, subunit ABC | SAS2310-12 | 100% | 99% | 99% | 99% | ⚫ | 100% | 99% | 99% |
| Haemolysin gamma, subunit AB | SAS1748-9 | 100% | 99% | 99% | 99% | ⚫ | 100% | - | 99% |
| Panton-Valentine leukocidin *lukS-PV* | MW1379 | - | - | - | - | - | ⚫ | - | - |
| Panton-Valentine leukocidin *lukF-PV* | MW1378 | - | - | - | - | - | ⚫ | - | - |
| Leukocidin family protein *lukXY* | SAS1924-5 | 100% | 97% | 99% | 99% | ⚫ | 100% | 86% | 97% |
| Toxic shock syndrome toxin | SA1819 | - | - | - | - | - | - | - | ⚫ |
| **Drug resistance** | | | | | | | | | |
| Metallo-beta-lactamase superfamily | SAS1905 | 100% | - | 97% | 97% | ⚫ | 100% | 97% | 97% |
| Beta-lactamase | SAS2333 | 99% | 98% | 96% | 98% | ⚫ | 99% | 95% | 98% |
| Metallo-beta-lactamase superfamily | SAS2500 | 100% | 99% | 99% | 99% | ⚫ | 100% | - | - |
| BlaI | pSAS17 | 100% | 100% | 99% | 100% | ⚫ | 66% | 99% | 66% |
| BlaR1 | pSAS18 | 99% | 99% | 95% | 99% | ⚫ | 67% | 96% | 67% |
| BlaZ | pSAS19 | 100% | 100% | 96% | 100% | ⚫ | - | 98% | - |
| Fosfomycin B | SAR2419 | - | 98% | - | 98% | - | - | ⚫ | 99% |
| Fusidic acid resistance protein | SAS0043 | 100% | - | - | - | ⚫ | - | - | - |
| **Metal resistance** | | | | | | | | | |
| CadCD | pSAS11-12 | 100% | 99% | 99% | 99% | ⚫ | - | - | - |

**Reference strains:** MW2 was isolated in 1998 and is a community acquired, methicillin resistant strain ([Baba, Takeuchi et al. 2002](#_ENREF_3)). MRSA252 was isolated in 1997 and is a hospital acquired methicillin resistant strain ([Holden, Feil et al. 2004](#_ENREF_18)). MSSA476 was isolated in 1998 and is a community acquired, invasive and generally susceptible strain ([Holden, Feil et al. 2004](#_ENREF_18)). N315 was isolated in 1982 and is a methicillin resistant strain ([Kuroda, Ohta et al. 2001](#_ENREF_21)).
