# Supplementary material for: Comparative genomics of non-pseudomonal bacterial species colonising paediatric cystic fibrosis patients
Source: PeerJ. 2015 Sep 15;3:e1223. doi: 10.7717/peerj.1223 (PMC4579023; doi:10.7717/peerj.1223)
Supplement: Table S5 [file peerj-03-1223-s005.docx]

| **Sample** | **SNPs** | **Within genes** | **Syn:NonSyn** | **Transition: Transversion** | **Indels** |
| --- | --- | --- | --- | --- | --- |
| **A8** | 20 | 18 | 0:18 | 6:14 | 4 |
| **A9** | 5 | 3 | 0:3 | 4:1 | 0 |
| **A10** | 5 | 5 | 0:5 | 2:3 | 0 |
| **A11** | 87 | 80 | 8:72 | 79:8 | 30 |
| **A12** | 6 | 6 | 3:3 | 4:2 | 2 |
| **A13** | 1 | 1 | 0:1 | 0:1 | 0 |
