# Supplementary material for: Comparative genomics of non-pseudomonal bacterial species colonising paediatric cystic fibrosis patients
Source: PeerJ. 2015 Sep 15;3:e1223. doi: 10.7717/peerj.1223 (PMC4579023; doi:10.7717/peerj.1223)
Supplement: Table S6 [file peerj-03-1223-s006.docx]

| **K279a locus** | **Details** | **Percent identity with K279a genomic locus** | | | | | |
| --- | --- | --- | --- | --- | --- | --- | --- |
|  |  | **B1** | **B4** | **B5** | **C11** | **A2** | ***D457*** |
| Smlt4476/5/4 (*smeABC*) | RND efflux pump, contributes to resistance to ciprofloxacin, gentamicin, tetracycline ([Chang et al. 2004](#_ENREF_5)) | 93% | 63% | 93% | 93% | 93% | 93% |
| Smlt4072/1/0 (*smeDEF*) | RND efflux pump, contributes to resistance to tetracycline, chloramphenicol, amikacin, erythromycin, norfloxacin, ofloxacin ([Alonso & Martínez 2000](#_ENREF_3)) | 94% | 90% | 95% | 94% | 96% | 96% |
| Smlt3170/1 (*smeGH*) | RND efflux pump, does not contribute to resistance in K279a ([Crossman et al. 2008](#_ENREF_6)) | 96% | 93% | 95% | 95% | 96% | 95% |
| Smlt4280/81 (*smeIJK*) | RND efflux pump, contributes to resistance to gentamicin, amikacin, tetracycline, minocycline, ciprofloxacin ([Crossman et al. 2008](#_ENREF_6)) | 95% | 92% | 95% | 95% | 95% | 95% |
| Smlt3788/7 (*smeMN*) | RND efflux pump, does not contribute to resistance in K279a ([Crossman et al. 2008](#_ENREF_6)) | 93% | 90% | 93% | 93% | 94% | 94% |
| Smlt3925/4 (*smeOP*) | RND efflux pump, does not contribute to resistance in K279a ([Crossman et al. 2008](#_ENREF_6)) | 95% | 89% | 95% | 95% | 95% | 95% |
| Smlt1830/1/2 (*smeVWX*) | RND efflux pump, does not contribute to resistance in K279a ([Crossman et al. 2008](#_ENREF_6)) | 96% | 93% | 96% | 96% | 96% | 96% |
| Smlt2201/2 (*smeYZ*) | RND efflux pump, contributes to resistance to gentamicin, kanamycin, amikacin, tobramycin ([Crossman et al. 2008](#_ENREF_6)) | 93% | 86% | 92% | 92% | 93% | 93% |
| Smlt1471 (*smrA*) | ABC efflux pump, contributes to resistance to ciprofloxacin, norfloxacin and tetracycline ([Al-Hamad et al. 2009](#_ENREF_1)) | 95% | 92% | 94% | 96% | 96% | 96% |
| Smlt1537/8/9 | Putative ABC-type tripartite efflux transporter | 94% | 70% | 93% | 93% | 94% | 94% |
| Smlt2642/3 | Putative ABC efflux transporter and MFP | 84% | - | 84% | - | 86% | 86% |
| Smlt0032 | Putative MFS-type tripartite efflux transporter | - | 70% | - | - | - | 70% |
| Smlt1528/9/30 (*emrAB*) | Putative MFS-type tripartite efflux transporter | 96% | 91% | 95% | 95% | 95% | 95% |
| Smlt2796/7/8 | Efflux pump, multidrug/fusaric acid resistance protein | 90% | - | - | 90% | 93% | 92% |
| Smlt2667 (*bla*_L1_) | Metallo-β-lactamase, contributes to resistance to penicillins, cephalosporins, carbapenems ([Avison et al. 2001](#_ENREF_4)) | 89% | 81% | 86% | 86% | 88% | 89% |
| Smlt3722 (*bla*_L2_) | Clavulanic acid sensitive β-lactamase, contributes to resistance to cephalosporins ([Avison et al. 2001](#_ENREF_4)) | 76% | 81% | 77% | 76% | 93% | 76% |
| Smlt0412/3 (*ampNG*) | β-lactamase induction signal transducer, essential for β-lactamase activity ([Huang et al. 2010](#_ENREF_7)). No BLAST hit in other strains to K279a *ampG.* See below for identity with *ampG* from strain D457. | 96% | 91% | 97% | 96% | 96% | 96% |
| SMD_0331/2 (*ampNG*) | β-lactamase induction signal transducer operon from strain D457. No BLAST hit in strain K279a to D457 version of *ampG.* | 94% | 91% | 95% | 98% | 99% | - |
| Smlt0115 (*ampC*) | Putative β-lactamase (cephalosporinase) | 93% | 79% | - | 88% | 95% | 93% |
| Smlt0347 | Putative metallo-β-lactamase | 91% | 87% | 91% | 92% | 92% | 92% |
| Smlt2120 (*aph(3’)-IIc*) | Aminoglycoside 3' phosphotransferase, contributes to resistance to butirosin, kanamycin, neomycin and paromomycin ([Okazaki & Avison 2007](#_ENREF_9)) | 89% | - | 83% | 84% | 87% | 88% |
| Smlt3615 (*aac(6’)-lz*) | Aminoglycoside 6'N acetyltransferase, contributes to resistance to amikacin, netilmicin, sisomicin and tobramycin (Li, 2003) | - | 84% | - | 90% | - | - |
| Smlt0191 | Putative aminoglycoside phosphotransferase | 91% | 80% | 88% | 92% | 91% | 91% |
| Smlt1669 | Putative aminoglycoside 2' N-acetyltransferase | - | - | - | - | - | - |
| Smlt2336 | Putative streptomycin 3' phosphotransferase/kinase | 89% | 85% | 87% | 90% | 90% | 89% |
| Smlt2125 (*spcN*) | Putative spectinomycin phosphotransferase | 89% | 78% | 87% | 87% | 89% | 89% |
| Smlt0620 (*cat*) | Putative chloramphenicol acetyltransferase | - | - | - | - | - | - |
| Smlt1071 (*qnrB*) | Quinolone target binding, contributes to resistance to levofloxacin, moxifloxacin, ciprofloxacin, ofloxacin, gatifloxacin ([Sánchez & Martínez 2010](#_ENREF_10)) | 92% | 92% | 92% | 95% | 94% | 94% |
| Smlt0818 (*ksgA*) | Putative kasuagamycin resistance protein | 95% | 93% | 95% | 96% | 96% | 96% |
| Smlt0821  (*ostA*) | Organic solvent tolerance protein | 92% | 88% | 95% | 92% | 92% | 92% |
| Smlt0269  (*ohr*) | Organic hydroperoxide | 97% | 96% | 97% | 97% | 97% | 97% |
| Smlt2409-12  (*merRTPA*) | Mercury resistance | - | 99% | - | 99% | - | 80% |
| Smlt2440-9  (*copLABMGCDF*) | Copper resistance | - | 99% | - | 99% | - | - |
| Smlt2691/2  (*copCD*) | Copper resistance | 87% | 75% | 82% | - | 89% | 87% |
| Smlt3690/1/2  (*copL2A2B2*) | Copper resistance | 92% | 88% | 89% | 90% | 92% | 91% |

Strain D457 is a clinical isolate displaying broad antibiotic susceptibility ([Alonso & Martínez 1997](#_ENREF_2); [Lira et al. 2012](#_ENREF_8))

**References**

Al-Hamad A, Upton M, and Burnie J. 2009. Molecular cloning and characterization of SmrA, a novel ABC multidrug efflux pump from *Stenotrophomonas maltophilia*. *Journal of Antimicrobial Chemotherapy* 64:731-734.

Alonso A, and Martínez JL. 1997. Multiple antibiotic resistance in *Stenotrophomonas maltophilia*. *Antimicrobial Agents and Chemotherapy* 41:1140-1142.

Alonso A, and Martínez JL. 2000. Cloning and characterization of SmeDEF, a novel multidrug efflux pump from *Stenotrophomonas maltophilia*. *Antimicrobial Agents and Chemotherapy* 44:3079-3086.

Avison MB, Higgins CS, von Heldreich CJ, Bennett PM, and Walsh TR. 2001. Plasmid location and molecular heterogeneity of the L1 and L2 β-lactamase genes of *Stenotrophomonas maltophilia*. *Antimicrobial Agents and Chemotherapy* 45:413-419.

Chang L-L, Chen H-F, Chang C-Y, Lee T-M, and Wu W-J. 2004. Contribution of integrons, and SmeABC and SmeDEF efflux pumps to multidrug resistance in clinical isolates of *Stenotrophomonas maltophilia*. *Journal of Antimicrobial Chemotherapy* 53:518-521.

Crossman L, Gould V, Dow JM, Vernikos G, Okazaki A, Sebaihia M, Saunders D, Arrowsmith C, Carver T, Peters N, Adlem E, Kerhornou A, Lord A, Murphy L, Seeger K, Squares R, Rutter S, Quail M, Rajandream M-A, Harris D, Churcher C, Bentley S, Parkhill J, Thomson N, and Avison M. 2008. The complete genome, comparative and functional analysis of *Stenotrophomonas maltophilia* reveals an organism heavily shielded by drug resistance determinants. *Genome Biology* 9:R74.

Huang Y-W, Lin C-W, Hu R-M, Lin Y-T, Chung T-C, and Yang T-C. 2010. AmpN-AmpG operon is essential for expression of L1 and L2 β-lactamases in *Stenotrophomonas maltophilia*. *Antimicrobial Agents and Chemotherapy* 54:2583-2589.

Lira F, Hernández A, Belda E, Sánchez MB, Moya A, Silva FJ, and Martínez JL. 2012. Whole-genome sequence of *Stenotrophomonas maltophilia* D457, a clinical isolate and a model strain. *Journal of Bacteriology* 194:3563-3564.

Okazaki A, and Avison MB. 2007. Aph(3')-IIc, an aminoglycoside resistance determinant from *Stenotrophomonas maltophilia*. *Antimicrobial Agents and Chemotherapy* 51:359-360.

Sánchez MB, and Martínez JL. 2010. SmQnr contributes to intrinsic resistance to quinolones in *Stenotrophomonas maltophilia*. *Antimicrobial Agents and Chemotherapy* 54:580-581.
