# Supplementary figures and images for: Comparative genomics of non-pseudomonal bacterial species colonising paediatric cystic fibrosis patients
Source: PeerJ. 2015 Sep 15;3:e1223. doi: 10.7717/peerj.1223 (PMC4579023; doi:10.7717/peerj.1223)

0 5,000 10,000 20,000 25,000

**pSAS1**

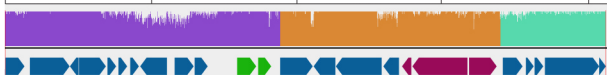

**pA1-1**

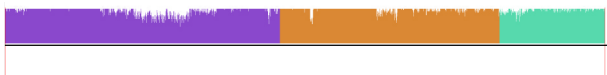

**pA3**

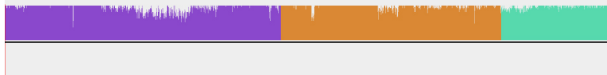

**pA4**

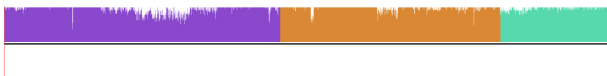

**pA5**

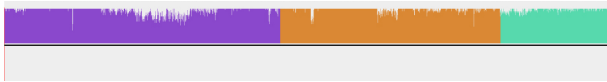

**pC9**

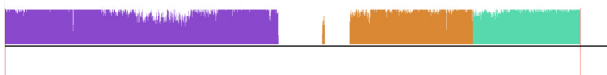

**pB6**

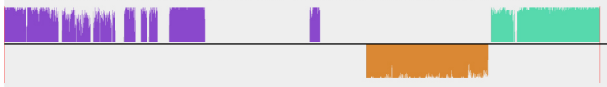

Supplement: Figure S1 — progressiveMauve (Darling, Mau & Perna, 2010) alignment of plasmid sequences from each isolate in comparison with the MSSA476 plasmid pSAS1. Similarity of sequence is indicated by coloured sections. β-lactamase genes are indicated in red, cadmium resistance genes are indicated in green. [file peerj-03-1223-s007.pdf]

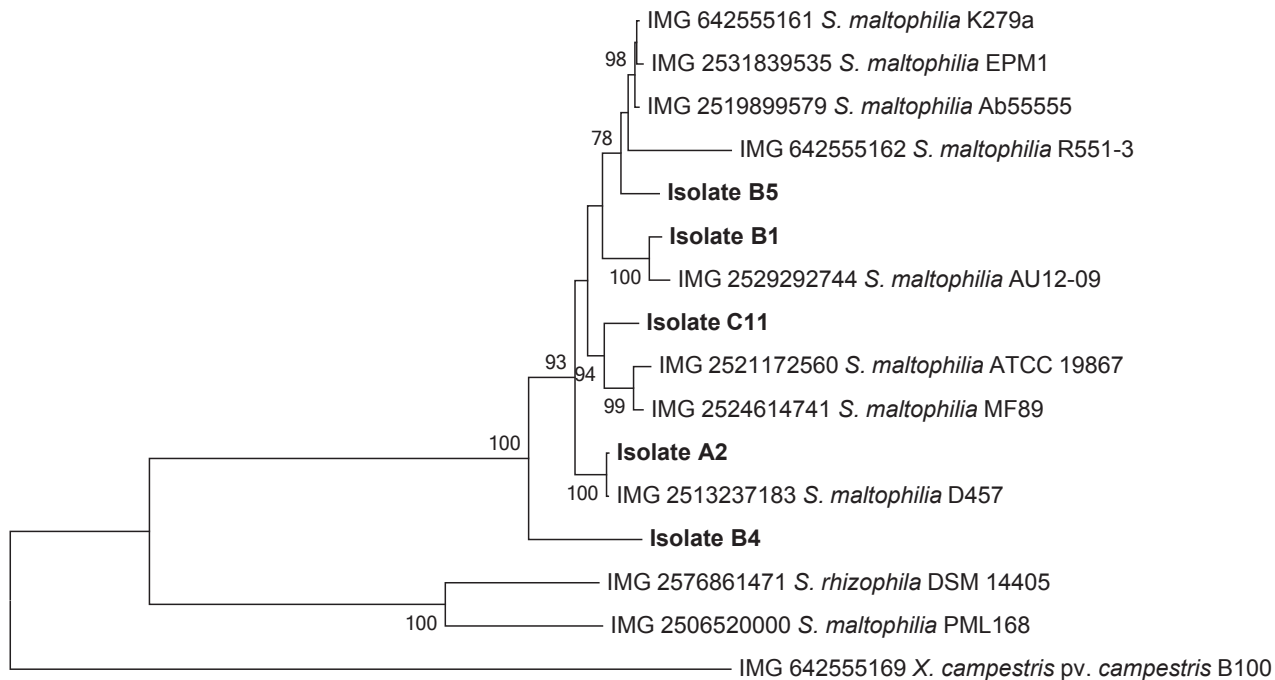

0.01

Supplement: Figure S2 — A maximum likelihood tree constructed using MEGA (Tamura et al., 2013) based on the concatenated alignment of 83 single copy genes generated using CheckM (Parks et al., 2015). The tree is drawn to scale, with branch lengths measured in the number of substitutions per site. Bootstrap (500 replicates) support values above 75% are shown. [file peerj-03-1223-s008.pdf]

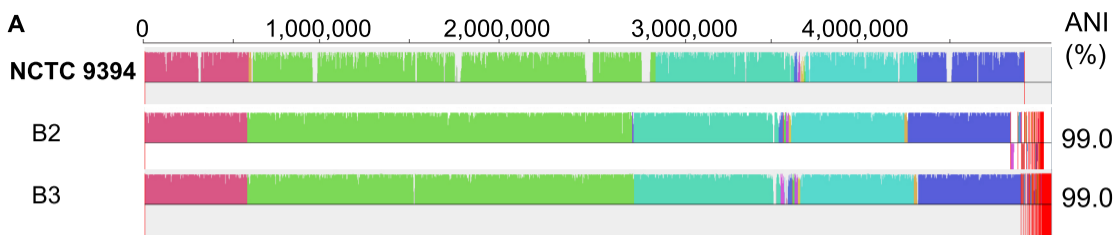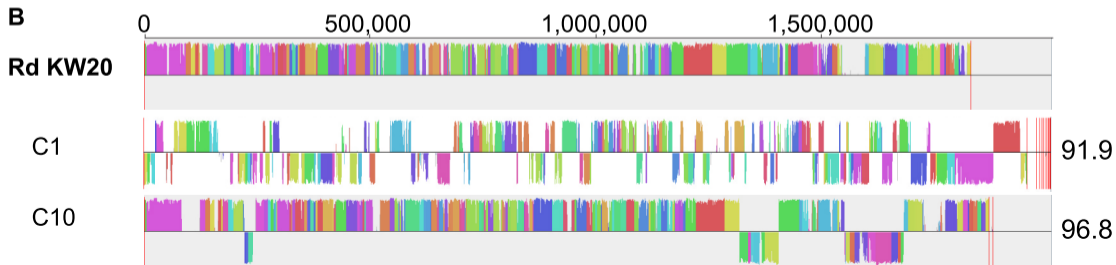

Supplement: Figure S3 — progressiveMauve (Darling, Mau & Perna, 2010) alignment of each strain with reference strain used for analysis. ANI calculated in comparison to the chosen reference strain. [file peerj-03-1223-s009.pdf]

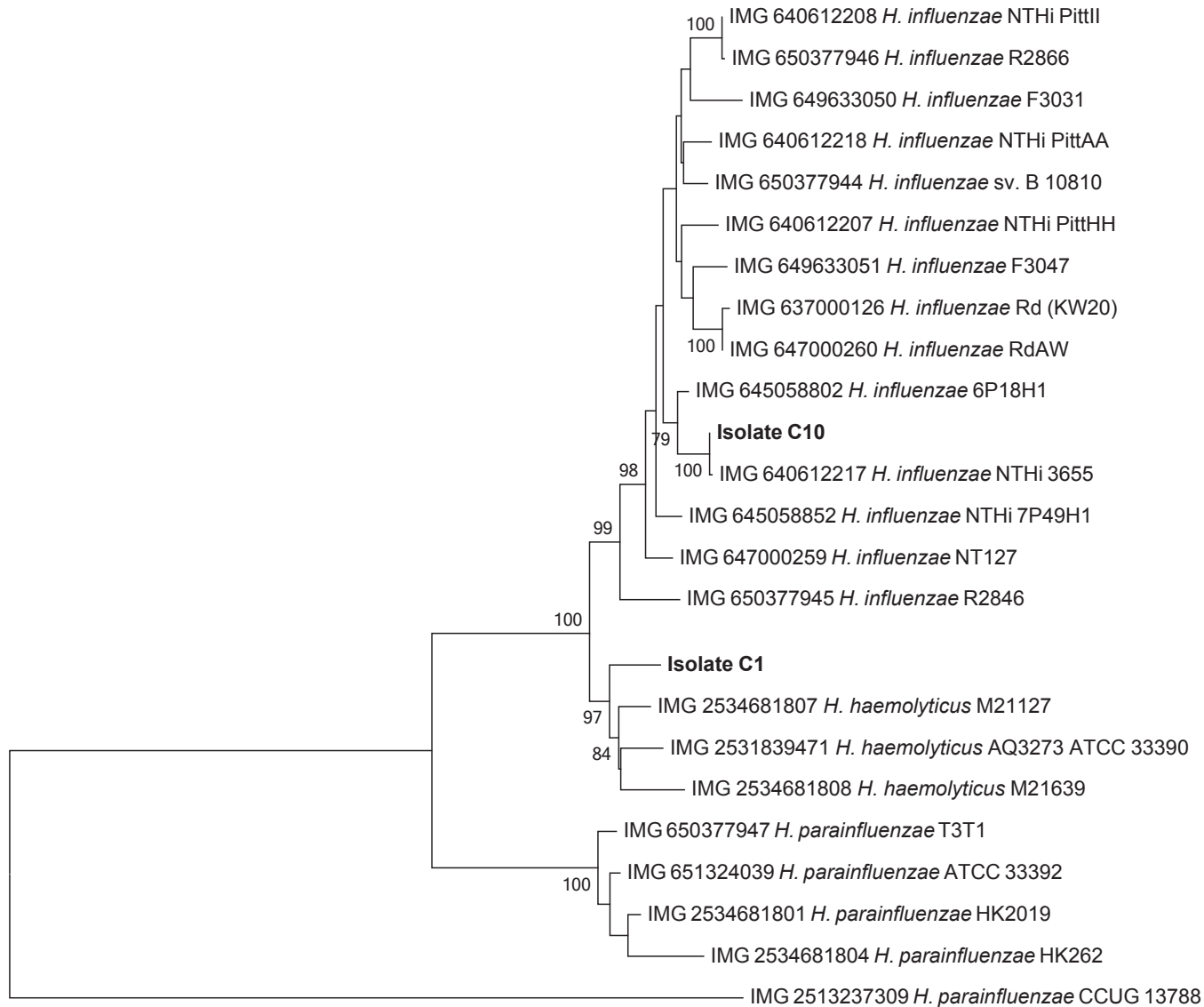

0.01

Supplement: Figure S5 — A maximum likelihood tree constructed using MEGA (Tamura et al., 2013) based on the concatenated alignment of 83 single copy genes generated using CheckM (Parks et al., 2015). The tree is drawn to scale, with branch lengths measured in the number of substitutions per site. Bootstrap (500 replicates) support values above 75% are shown. [file peerj-03-1223-s011.pdf]
