# Supplementary material for: Comparative genomics of non-pseudomonal bacterial species colonising paediatric cystic fibrosis patients
Source: PeerJ. 2015 Sep 15;3:e1223. doi: 10.7717/peerj.1223 (PMC4579023; doi:10.7717/peerj.1223)
Supplement: Figure S4 — Alignment generated using Easyfig (Sullivan et al., 2011). [file peerj-03-1223-s010.pdf]

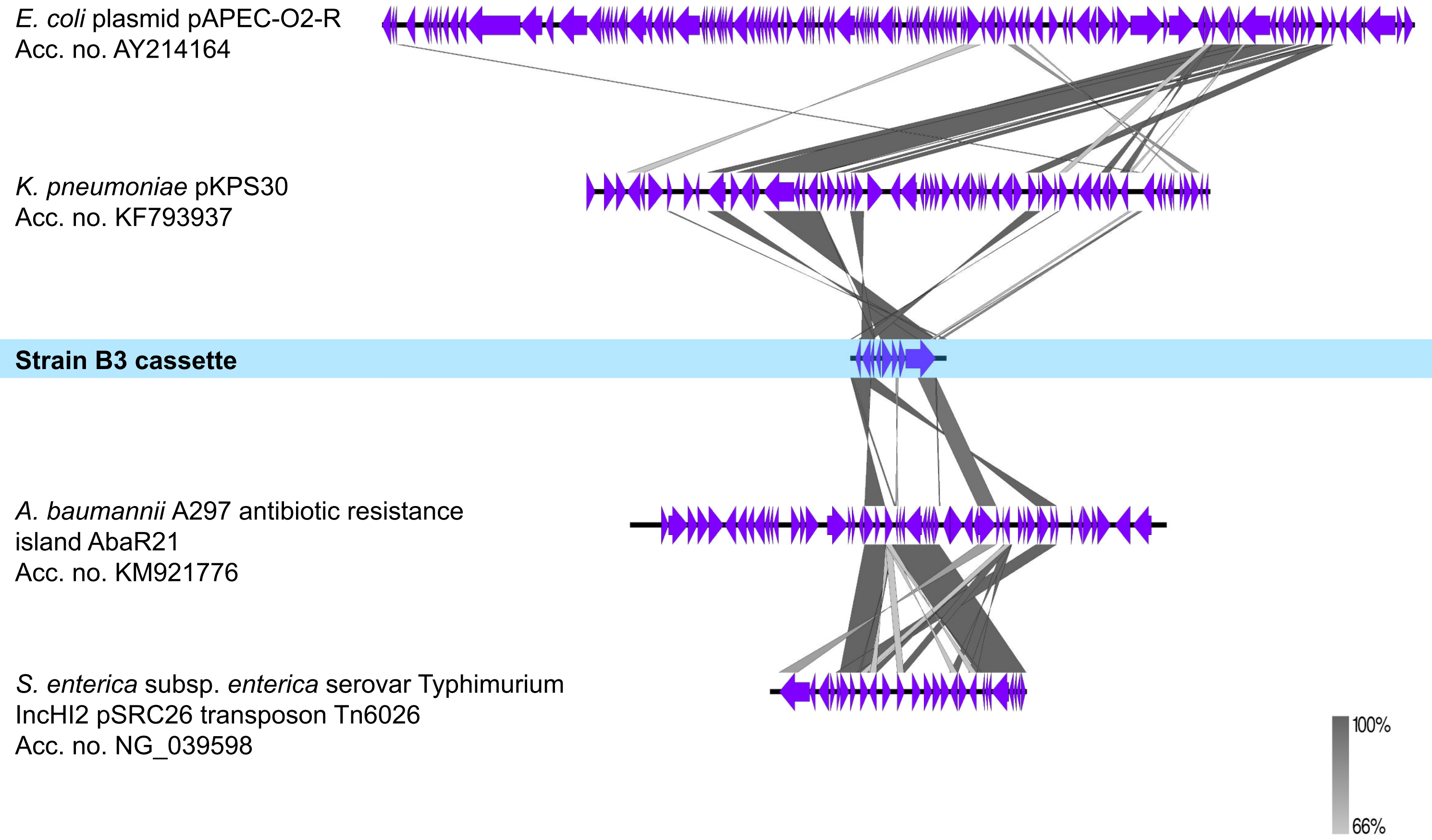

E. coli plasmid pAPEC-O2-R
Acc. no. AY214164
K. pneumoniae pKPS30
Acc. no. KF793937
Strain B3 cassette
A. baumannii A297 antibiotic resistance
island AbaR21
Acc. no. KM921776
S. enterica subsp. *enterica* serovar Typhimurium
IncHI2 pSRC26 transposon Tn6026
Acc. no. NG_039598
100%
66%
